# Higher-Order Oligomerization Targets Plasma Membrane Proteins and HIV Gag to Exosomes 

**DOI:** 10.1371/journal.pbio.0050158

**Published:** 2007-06-05

**Authors:** Yi Fang, Ning Wu, Xin Gan, Wanhua Yan, James C Morrell, Stephen J Gould

**Affiliations:** Department of Biological Chemistry, Johns Hopkins University School of Medicine, Baltimore, Maryland, United States of America; University of California San Francisco, United States of America

## Abstract

Exosomes are secreted organelles that have the same topology as the cell and bud outward (outward is defined as away from the cytoplasm) from endosome membranes or endosome-like domains of plasma membrane. Here we describe an exosomal protein-sorting pathway in Jurkat T cells that selects cargo proteins on the basis of both higher-order oligomerization (the oligomerization of oligomers) and plasma membrane association, acts on proteins seemingly without regard to their function, sequence, topology, or mechanism of membrane association, and appears to operate independently of class E vacuolar protein-sorting (VPS) function. We also show that higher-order oligomerization is sufficient to target plasma membrane proteins to HIV virus–like particles, that diverse Gag proteins possess exosomal-sorting information, and that higher-order oligomerization is a primary determinant of HIV Gag budding/exosomal sorting. In addition, we provide evidence that both the HIV late domain and class E VPS function promote HIV budding by unexpectedly complex, seemingly indirect mechanisms. These results support the hypothesis that HIV and other retroviruses are generated by a normal, nonviral pathway of exosome biogenesis.

## Author Summary

Exosomes are small, secreted organelles with the same topology as the cell and a similar size and composition as retrovirus particles. Based on these similarities, we proposed that retroviruses are, at their most fundamental level, exosomes. Little is known about the mechanisms of exosome biogenesis. We show here that higher-order oligomerization and plasma membrane binding are sufficient to target proteins into both exosomes and HIV virus-like particles. We also find that the HIV protein Gag, which possesses these general exosomal sorting elements, requires only these elements to bud from human cells. Others have proposed that the HIV p6 domain and the host class E vacuolar protein-sorting (VPS) machinery play direct, essential, and mechanistic roles in HIV budding. However, we show here that p6-deficient HIV can bud from cells at normal levels and that both p6-deficient HIV and exosomes can bud independently of class E VPS function. Thus, it appears that exosome biogenesis pathways mediate the budding of HIV from cells, whereas the HIV p6 domain and the class E VPS machinery promote budding indirectly.

## Abstract 3

Experiments in Jurkat T cells suggest that HIV and other retroviruses may co-opt the normal nonviral pathway of exosome biogenesis.

## Introduction

Exosomes are secreted organelles that have the same topology as the cell and a diameter of approximately 50–150 nm, though larger exosomes have also been reported [1–3]. Exosome content varies considerably, but commonly includes tetraspanins, integrins, major histocompatibility complex proteins, cytosolic chaperones, cholesterol, and glycosphingolipids [2,4]. Many animal cell types secrete exosomes, and exosome-mediated signaling has been implicated in antigen presentation, morphogenesis, sperm maturation, and cancer–host interactions [2,4–6]. Less is known about the mechanisms of exosome biogenesis.

Cells can generate exosomes by either of two modes, immediate or delayed. The immediate mode of exosome biogenesis occurs at the cell surface and involves outward vesicle budding (outward is defined as away from the cytoplasm) from endosome-like domains of the plasma membrane, domains we refer to as ELDs [3]. In contrast, the delayed mode of exosome biogenesis begins with outward vesicle budding at the limiting membrane of endosomes, generating vesicle-laden endosomes, typically referred to as multivesicular bodies (MVBs) [2,4]. If an MVB fuses with the plasma membrane, its internal vesicles are released as exosomes. However, MVBs can also fuse with lysosomes, leading to vesicle and cargo destruction [7]. Some cell types, such as T cells, prefer to make exosomes by the immediate mode, whereas other cell types, such as macrophages, prefer to make exosomes via the delayed mode. Interestingly, human immunodeficiency virus (HIV) particles bud from these two cell types at the same sites as exosomes, have the same topology as exosomes, have a similar size as exosomes, and are enriched in the same molecules as exosomes [3,8–13]. These and other observations indicate that there might be a mechanistic relationship between retrovirus budding and exosome biogenesis [11].

A major step in understanding the biogenesis of any organelle is to identify and characterize the *cis*-acting signals that target proteins to the organelle. Here we show that higher-order oligomerization and plasma membrane association target proteins to sites of exosome budding and into exosomes. Proteins can be directed into this exosomal protein sorting pathway by (1) exposing cell surface proteins to exogenous cross-linking agents (e.g., primary and secondary antibodies), (2) appending plasma membrane anchors to highly oligomeric cytoplasmic proteins, or (3) adding multiple homo-oligomerization domains to intracellular acylated proteins. The class E vacuolar protein-sorting (VPS) proteins are thought to drive outward vesicle budding [14], but we find that inhibiting class E VPS function does not block exosome budding or the oligomerization-induced exosomal protein-sorting pathway. We also find that retroviral Gag proteins are sorted to ELDs and exosomes, that exosomal targeting information directs proteins to sites of HIV Gag budding and onto HIV virus–like particles (VLPs), that higher-order oligomerization is a primary determinant of HIV Gag budding, that p6-deficient HIV can bud independently of class E VPS function, and that acquisition of exosomal sorting information is sufficient to induce the budding of a yeast long terminal repeat (LTR) retrotransposon. Taken together, these results support the hypothesis that retroviral budding is a form of exosome biogenesis [11].

## Results

### Higher-Order Oligomerization Targets Plasma Membrane Proteins to ELDs and Exosomes

Jurkat T cells bud exosomes from ELDs [3]. ELDs are often clustered at a single pole in Jurkat T cells [3], resembling the surface protein “caps” that form after antibody-induced oligomerization of leukocyte plasma membrane proteins [15,16]. To determine whether there is any relationship between exosomal protein sorting and surface protein capping, we marked ELDs by pulse-labeling Jurkat T cells with an exosomal lipid, N-Rh-PE [3], chilled the cells to 4 °C, then incubated the cells with monoclonal (bivalent) immunogammaglobulin (IgG) antibodies specific for known T cell plasma membrane proteins, washed the cells, and incubated the cells with fluorescein isothiocyanate (FITC)-labeled polyclonal anti-mouse IgG antibodies, all on ice. Half the cells were fixed immediately, half were incubated at 37 °C for 1–2 h and then fixed, and both were examined by fluorescence microscopy (Figure 1A–1X). At time 0, the plasma membrane markers CD43, CD45, and CD59 showed no significant enrichment at ELDs. In contrast, cells incubated at 37 °C sorted these antibody–antigen complexes to ELDs. Similar results were observed for CD4, CD5, CD28, CD31, CD38, CD55, CD62L, CD98, CD99, and PrP (unpublished data), as well as in cells expressing a different marker for ELDs, AIP1/VPS31-DsRED (unpublished data) [3].

**Figure 1:**
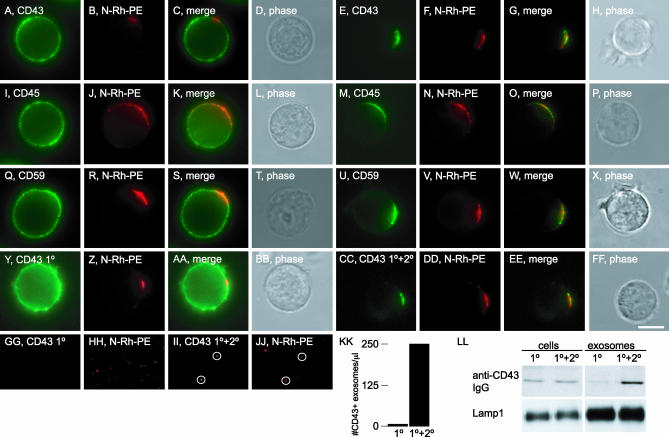
Higher-Order Oligomerization Targets Proteins to ELDs (A–X) N-Rh-PE–labeled Jurkat T cells were incubated with monoclonal antibodies specific for (A–H) CD43, (I–P) CD45, or (Q–X) CD59 and FITC-labeled polyclonal anti-mouse antibodies, on ice, and then either (A–D, I–L, and Q–T) fixed or (E–H, M–P, and U–X) incubated at 37 °C for 2 h and then fixed. (Y–LL) Higher-order oligomerization targets CD43 to exosomes. (Y–FF) N-Rh-PE–labeled Jurkat T cells were incubated with FITC-labeled monoclonal antibodies specific for CD43, then incubated with either (Y–BB) buffer alone or (CC–FF) unlabeled polyclonal rabbit anti-mouse IgG antibodies, all on ice. Cells were incubated for 24 h at 37 °C and then examined. (GG–KK) Fluorescence microscopy of exosomes from cells treated with (GG and HH) primary antibodies only or (II and JJ) primary and secondary antibodies. (KK) Numbers of FITC-positive exosomes secreted by cells exposed to (left bar) primary antibodies only or (right bar) primary and secondary antibodies. Bar indicates 10 μm. (LL) Samples from Jurkat cells incubated with either mouse anti-CD43 IgG only (1°) or with mouse anti-CD43 IgG and anti-mouse IgG (1° + 2°) were blotted with anti-mouse antibodies to determine the amount of CD43 secreted on exosomes, and anti-Lamp1 antibodies to determine the level of exosome secretion. Bar indicates 10 μm.

Surface protein “capping” has been reported to require the oligomerization of oligomers [15,16], which we refer to as higher-order oligomerization. To determine whether higher-order oligomerization was sufficient for targeting CD43 to ELDs and exosomes, N-Rh-PE–labeled Jurkat T cells were chilled to 4 °C, incubated with FITC-labeled monoclonal (IgG) anti-CD43 antibodies (on ice), separated into two equal fractions, and incubated with either a mock solution or unlabeled polyclonal rabbit anti-mouse IgG antibodies, all on ice, followed by incubation overnight at 37 °C. Fluorescence microscopy of the two cell populations revealed that CD43 was sorted to ELDs only after the addition of polyclonal secondary antibodies (Figure 1Y–1FF). Thus, higher-order oligomerization is both necessary and sufficient to target CD43 to ELDs.

ELDs are sites of exosome budding, and we next tested whether higher-order oligomerization is sufficient to induce a protein's budding/exosomal sorting (we define the term “budding” to mean the secretion of a molecule on sedimentable vesicles that have the general properties of exosomes/VLPs). N-Rh-PE–labeled T cells that had been exposed to only monoclonal anti-CD43 antibodies secreted N-Rh-PE–labeled exosomes that were mostly lacking the FITC-labeled CD43–antibody complex. In contrast, exosomes secreted by cells exposed to both the FITC-labeled monoclonal anti-CD43 antibody and the polyclonal anti-mouse IgG antibodies were approximately 30-fold more likely to carry detectable levels of the FITC-labeled CD43–antibody complex (Figure 1GG–1KK). In paralogous experiments using unlabeled antibodies, immunoblot analysis of cell and exosome lysates revealed that higher-order oligomerization induces an approximately 10-fold increase in the amount of CD43–antibody complex secreted from the cell in exosomes (Figure 1LL). This increase in exosomal CD43 did not appear to reflect a general increase in exosome biogenesis, because the levels of exosomal Lamp1 were unaffected by these manipulations.

### A Plasma Membrane Anchor Targets Highly Oligomeric Cytoplasmic Proteins to ELDs and Exosomes

The hypothesis that higher-order oligomerization is sufficient to target plasma membrane proteins to ELDs and exosomes has a clear corollary: a plasma membrane anchor should be sufficient to target highly oligomeric, cytoplasmic proteins to ELDs and exosomes. To explore this possibility, we used the yeast protein TyA, which assembles into large oligomeric structures in the cytoplasm of yeast cells [17,18]. The suitability of TyA for these studies is enhanced by the fact that it is derived from the Saccharomyces cerevisiae Ty1 LTR retrotransposon, which accumulates in the cytoplasm, does not bud from cells, is not infectious, and replicates in an organism that is devoid of retroviruses.

When expressed in Jurkat T cells, wild-type (WT) TyA (tagged with green fluorescent protein [GFP] at its C-terminus) accumulated in the cytoplasm and showed no enrichment at ELDs (Figure 2A–2D). In contrast, AcylTyA-GFP, which contains a 10 amino acid–long acylation tag at its N-terminus (designed to confer myristoylation at Gly2, palmitoylation at Cys3, and targeting of the protein to the plasma membrane [19]), co-localized at ELDs with the exosomal markers N-Rh-PE and surface CD63 (Figure 2E–2L). A mutant form of AcylTyA-GFP lacking the putative acylation sites, Acyl(G2A,C3A)TyA-GFP (containing alanine residues at positions 2 and 3 of the tag), was not sorted to ELDs and instead accumulated in the cytoplasm of Jurkat T cells (Figure 2M–2P).

**Figure 2:**
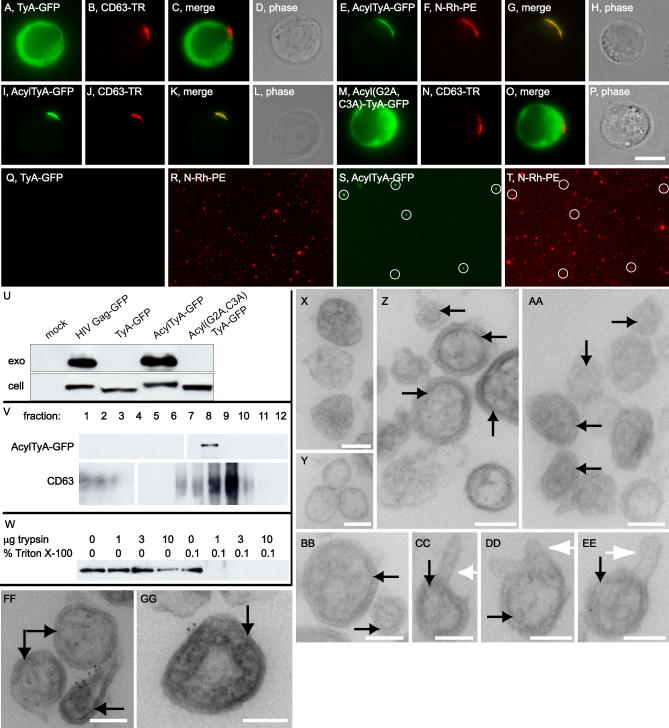
A Plasma Membrane Anchor Targets TyA to ELDs and Exosomes (A–P) Fluorescence microscopy of (A–D) Jurkat T cells expressing TyA-GFP, fixed and stained with antibodies to detect surface CD63; (E–H) N-Rh-PE–labeled Jurkat T cells expressing AcylTyA-GFP; (I–L) Jurkat T cells expressing AcylTyA-GFP, fixed and stained with antibodies to detect surface CD63; (M–P) Jurkat T cells expressing Acyl(G2A,C3A)TyA-GFP, fixed and stained with antibodies to detect surface CD63. Bar indicates 10 μm. (Q–T) Fluorescence microscopy of exosomes secreted by N-Rh-PE–labeled Jurkat T cells expressing (Q and R) TyA-GFP or (S and T) AcylTyA-GFP. White circles mark exosomes that contain both N-Rh-PE and AcylTyA-GFP. (U) Anti-GFP immunoblot of exosomes (exo) and cell lysates (cell) prepared from Jurkat T cells (mock) and Jurkat T cells expressing HIV Gag-GFP, TyA-GFP, AcylTyA-GFP, or Acyl(G2A,C3A)TyA-GFP. (V) Exosomes secreted by Jurkat T cells expressing AcylTyA-GFP were purified by sucrose density centrifugation, fractions were collected from the bottom of the gradient, and equal amounts of each fraction were examined by immunoblot using antibodies specific for (upper panel) GFP and (lower panel) the exosomal marker CD63. Fractions 1–12 were of the densities 1.34, 1.33, 1.33, 1.32, 1.24, 1.20, 1.16, 1.13, 1.11, 1.10, and 1.09 g/ml, respectively. (W) Anti-GFP immunoblot of exosomes collected from Jurkat T cells expressing AcylTyA-GFP and incubated with different amounts of trypsin in the absence or presence of 0.1% Triton X-100. (X–GG) Immunoelectron microscopy of N-Rh-PE–labeled Jurkat T cells expressing (X and Y) Acyl(G2A,C3A)TyA or (Z–GG) AcylTyA. Black arrows denote electron-dense lamina under the membrane of exosomes secreted by cells expressing AcylTyA, white arrows denote exosome protrusions. (FF and GG) Six-nanometer immunogold is directed against rhodamine of N-Rh-PE. Bar indicates 100 nm.

To determine whether the sorting to ELDs correlated with secretion in exosomes, we collected exosomes from N-Rh-PE–labeled Jurkat T cells expressing either TyA-GFP or AcylTyA-GFP, bound them to glass, and examined them by fluorescence microscopy. Jurkat T cells failed to secrete TyA-GFP from the cell in exosomes (Figure 2Q and 2R), but did secrete AcylTyA-GFP from the cell in N-Rh-PE–containing exosomes (Figure 2S and 2T). In a separate experiment, we generated cell and exosome lysates from Jurkat T cells either mock-transfected or transfected with plasmids designed to express HIV Gag-GFP (which is sufficient for budding [20] and buds from the cell in exosomes [3]), TyA-GFP, AcylTyA-GFP, and Acyl(G2A,C3A)TyA-GFP, and then subjected these to immunoblot analysis. Using a constant ratio of cell lysate:exosome lysate, we observed that AcylTyA-GFP was selectively secreted from the cell in exosomes (Figure 2U), as was HIV Gag-GFP.

We further purified exosomes from these cells by sucrose density flotation gradient centrifugation and assayed fractions across the gradient by immunoblot using antibodies specific for GFP and for CD63, a known exosomal marker. AcylTyA-GFP co-fractionated with CD63, providing further evidence that it was secreted from the cell in exosomes (Figure 2V). Exosomes collected from these cells were also subjected to protease protection experiments. AcylTyA-GFP was degraded far more extensively in the presence of trypsin and Triton X-100 than when exposed to trypsin alone (Figure 2W), indicating that AcylTyA-GFP was located in the lumen of the exosomes.

Electron microscopy experiments provided additional evidence that the acylation tag is sufficient to induce the budding of TyA. TyA is known to form electron-dense protein complexes [17,18], and thus, cells expressing an exosomal form of TyA would be expected to secrete exosomes that contain an electron-dense lamina under their membrane. Jurkat T cells normally secrete exosomes that lack an electron-dense lamina under their membrane [3], and this was observed for exosomes secreted by cells expressing unmodified, non-exosomal TyA (Figure 2X and 2Y). In contrast, cells expressing AcylTyA secreted exosomes that resembled retroviral VLPs in that they had an electron-dense lamina under their membrane (Figure 2Z–2GG). These AcylTyA-containing exosomes varied significantly in size, from approximately 50-nm diameter to approximately 250-nm diameter, and were typically of spheroid morphology, though some possessed short membrane protrusions (Figure 2CC–2EE). AcylTyA-containing exosomes also labeled for N-Rh-PE, shown here using 6-nm immunogold (Figure 2FF and 2GG).

### Retroviral Gag Proteins Are Exosomal Cargoes

Like the exosomal cargoes described above, the Gag proteins of the Orthoretroviridae (true retroviruses) are known to bind the plasma membrane and assemble into higher-order oligomeric complexes [21]. Therefore, if higher-order oligomerization and plasma membrane binding are sufficient for exosomal targeting, orthoretroviral Gag proteins should be sorted to ELDs and exosomes. Equine infectious anemia virus (EIAV), human T-cell lymphotropic virus-1 (HTLV-1), murine leukemia virus (MLV), Rous sarcoma virus (RSV), Mason-Pfizer monkey virus (MPMV), and human endogenous retrovirus-K (HERV-K) represent five major families of the Orthoretroviridae. The Gag protein from each of these viruses was expressed in Jurkat T cells as a C-terminally GFP-tagged protein, and in each case, the Gag-GFP protein was sorted to ELDs. This is shown here by their co-localization with surface CD63, one exosomal marker (Figure 3), as well as by their co-localization with the ELD/exosome markers N-Rh-PE and AIP1-DsRED (unpublished data). As a negative control, we followed the sorting of the Gag protein from simian foamy virus (SFV), a representative of the Spumaviridae. The Spumaviridae are the viruses most closely related to the Orthoretroviridae, but SFV budding is mediated by its envelope protein rather than its Gag protein [21]. SFV Gag-GFP was not sorted to ELDs (Figure 3CC–FF).

**Figure 3:**
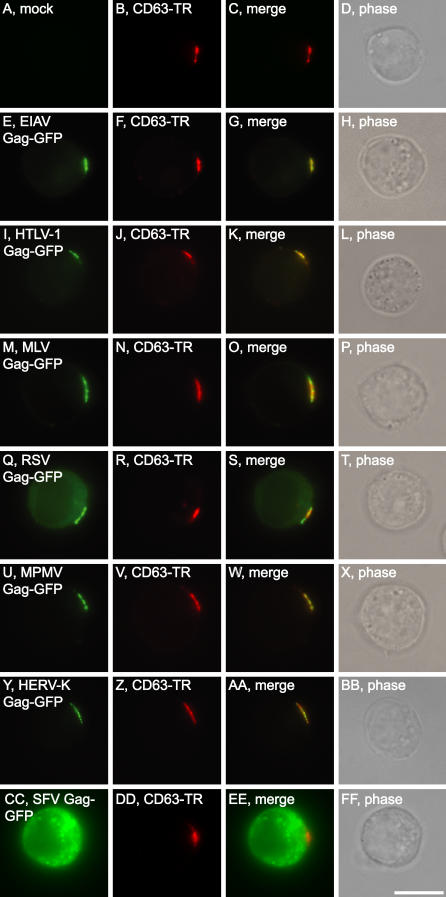
Jurkat T Cells Selectively Sort Orthoretroviral Gag Proteins to ELDs Fluorescence microscopy of Jurkat T cells (A–D) untransfected (mock) and (E–FF) transfected with plasmids designed to express GFP-tagged Gag proteins from (E–H) EIAV, (I–L) HTLV-1, (M–P) MLV, (Q–T) RSV, (U–X) MPMV, (Y–BB) HERV-K, and (CC–FF) SFV, each stained for surface CD63. Bar indicates 10 μm.

To determine whether these Gag proteins were secreted from the cell in exosomes, exosomes were collected from Jurkat T cells that had been pulse labeled with N-Rh-PE and transfected with expression vectors for each Gag protein. Two days later, exosomes were collected, bound to glass, and visualized by fluorescence microscopy. Mock-transfected cells secreted exosomes that lacked GFP, as expected (Figure 4A and 4B). In contrast, Jurkat T cells secreted each of the orthoretroviral Gag proteins in discrete particles, nearly all of which were also labeled with the exosomal lipid N-Rh-PE (Figure 4C–4N). SFV Gag-GFP could not be detected in exosomes (Figure 4O and 4P). Immunoblot analysis confirmed these observations (Figure 4Q).

**Figure 4:**
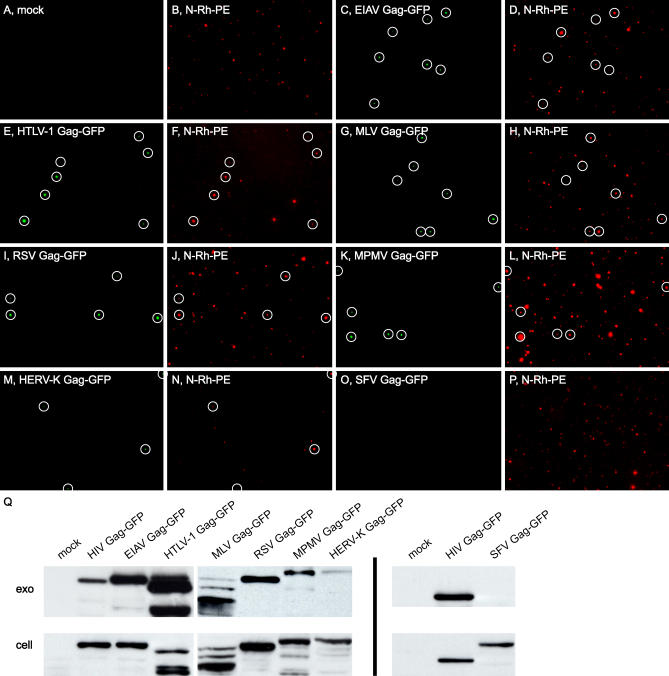
Jurkat T Cells Selectively Sort Orthoretroviral Gag Proteins to Exosomes (A–P) Fluorescence microscopy of exosomes secreted by N-Rh-PE–labeled Jurkat T cells (A and B) untransfected (mock) or transfected with plasmids that express GFP-tagged Gag proteins from (C and D) EIAV, (E and F) HTLV-1, (G and H) MLV, (I and J) RSV, (K and L) MPMV, (M and N) HERV-K, or (O and P) SFV. White circles denote vesicles containing the Gag-GFP protein, almost all of which co-localize with the exosomal marker N-Rh-PE. (Q) Anti-GFP immunoblots of (upper panel) exosomes (exo) and (lower panel) cell lysates (cell) (using the same exosome:cell ratio for all samples) from mock-transfected (mock) Jurkat T cells and Jurkat T cells expressing GFP-tagged Gag proteins from HIV, EIAV, HTLV-1, MLV, RSV, MPMV, HERV-K, and SFV. Bar indicates 10 μm.

### Higher-Order Oligomerization Targets a Plasma Membrane Protein to HIV VLPs

If retroviral budding is a form of exosome biogenesis, then higher-order oligomerization and plasma membrane binding should target proteins to HIV VLPs (the Gag-containing vesicles secreted by Gag-expressing cells). To test this prediction, we expressed HIV Gag-DsRED in Jurkat T cells and exposed these cells to FITC-labeled monoclonal anti-CD43 antibodies. The sample was then split in half, incubated with either mock solution or polyclonal anti-mouse IgG antibodies, incubated overnight at 37 °C, and examined by fluorescence microscopy. For cells exposed to only primary anti-CD43 IgG, the FITC-labeled antibody–CD43 complex showed little if any co-localization with HIV Gag-DsRED at ELDs (Figure 5A–5D). In contrast, the higher-order oligomerization of CD43 induced by adding polyclonal anti-mouse IgG antibodies caused the co-localization of CD43–antibody complexes with HIV Gag-DsRED at ELDs (Figure 5E–5H). Moreover, the Gag-DsRED-expressing cells that were exposed to both the FITC-labeled anti-CD43 IgG and polyclonal anti-mouse IgG antibodies secreted significant numbers of Gag-DsRED-containing VLPs that also carried FITC-labeled CD43–antibody complexes (Figure 5I–5K).

**Figure 5:**
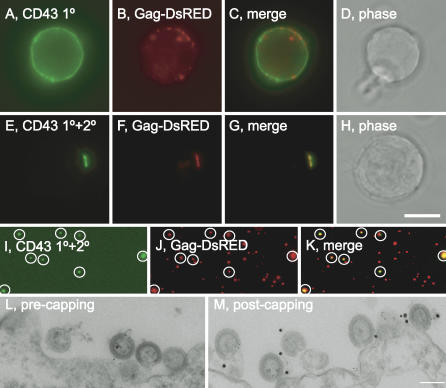
Higher-Order Oligomerization Targets CD43 to HIV VLPs (A–H) Jurkat T cells expressing HIV Gag-DsRed were labeled with FITC-conjugated mouse anti-CD43 antibodies and either (A–D) buffer or (E–H) polyclonal rabbit anti-mouse IgG antibodies, grown for 24 h at 37 °C, and examined by fluorescence microscopy of. Bar indicates 10 μm. (I–K) Fluorescence microscopy of exosomes secreted by cells expressing HIV Gag-DsRED and incubated with FITC-primary and secondary antibodies to CD43. White circles mark exosomes that contain both CD43-antibody complexes and HIV Gag-DsRED. (L and M) Immunoelectron microscopy of N-Rh-PE–labeled Jurkat T cells expressing untagged, full-length HIV Gag, incubated with primary and secondary antibodies to CD43 and then either (L) fixed or (M) incubated at 37 °C for 2 h and then fixed. Samples were then incubated with immunogold to detect (6 nm) N-Rh-PE and (18 nm) CD43. Bar indicates 100 nm.

To test these conclusions in greater detail, we performed similar experiments on N-Rh-PE–labeled Jurkat T cells that expressed untagged, full-length HIV Gag. Using 6-nm immunogold to detect N-Rh-PE, 18-nm immunogold to detect CD43, and the electron-dense Gag core to detect sites of VLP budding and VLPs, we quantified the amount of CD43 present on budding VLPs following CD43 dimerization (monoclonal antibodies only) or higher-order oligomerization. In cells exposed to only primary anti-CD43 antibodies, the levels of CD43–antibody complexes on emerging HIV VLPs were relatively low (Figure 5L; CD43-gold detected on 7/110 Gag-containing vesicles, with an average of 0.10 ± 0.04 gold grains/Gag-containing vesicle (± standard error)). In contrast, higher-order oligomerization of CD43 caused a nearly 10-fold increase in the amount of CD43–antibody complexes on budding HIV VLPs (Figure 5M; CD43-gold detected on 70/119 Gag-containing vesicles, with an average of 0.94 ± 0.09 gold grains/Gag-containing vesicle). The greater labeling for N-Rh-PE than CD43 is likely due to the generally better labeling for small immunogold conjugates as well as the addition of a significant quantity of N-Rh-PE to the cells.

### Higher-Order Oligomerization Drives the Budding of HIV Gag

The oligomerization-induced sorting of plasma membrane proteins to ELDs, exosomes, and VLPs led us to examine the sorting information in HIV Gag, the key budding factor in HIV. HIV Gag clearly has the expected properties of an exosomal cargo in that it assembles into highly oligomeric core particles of greater than 100 mDa (up to 5,000 polypeptides/core), is anchored in the inner leaflet of the plasma membrane via an N-terminal myristoyl moiety [22–24], and is sorted to ELDs and exosomes by Jurkat T cells [3]. Moreover, it is known that the budding of HIV Gag and virus is blocked by mutations that prevent its anchoring in the plasma membrane [24] or disrupt either of its two major oligomerization domains [25–30], which are located in its capsid (CA) and nucleocapsid (NC) domains, respectively (Figure 6A). On the other hand, the prevailing hypothesis is that Gag and virus budding is driven by (1) the actions of its late domain, the PTAP sequence located in the Gag p6 domain (Figure 6A), and (2) the specific architecture of the homo-oligomeric Gag core complex, which is thought to promote budding by deforming the cell membrane [31]. This hypothesis is supported by several lines of evidence, most notably (1) reduced budding of HIV late domain mutants (in certain cell types), (2) physical interaction between the late domain motif (and other p6 sequences) with certain class E VPS proteins, and (3) reduced HIV budding in cells with impaired class E VPS function [14,31–37].

**Figure 6:**
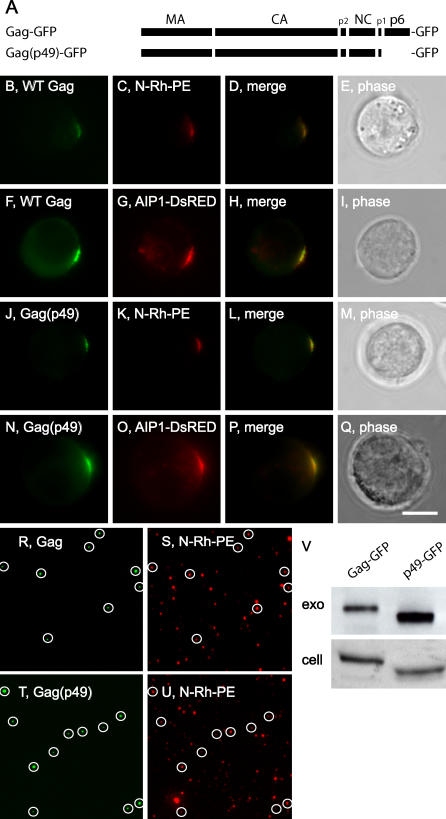
HIV Gag Is Sorted to ELDs and Exosomes Independently of Its p6 Domain (A) Line diagram of HIV Gag showing the relative positions and lengths of its MA (matrix), CA (capsid), p2, NC (nucleocapsid), p1, and p6 domains, as well as the absence of p6 from HIV Gag(p49)-GFP. (B–Q) Fluorescence micrographs of Jurkat T cells expressing (B–I) HIV Gag-GFP and (J–Q) HIV Gag(p49)-GFP, that had either been (B–E and J–M) incubated previously with the exosomal lipid N-Rh-PE or (F–I and N–Q) co-transfected with a plasmid that expresses AIP1-DsRED. (R–U) Fluorescence micrographs of exosomes collected from N-Rh-PE–labeled Jurkat T cells expressing either (R and S) HIV Gag-GFP or (T and U) HIV Gag(p49)-GFP. White circles mark exosomes that contain both N-Rh-PE and Gag-GFP or Gag(p49)-GFP. (V) Anti-Gag immunoblot of exosomes (exo) and cell lysates (cell) from Jurkat T cells expressing either (left lanes) HIV Gag-GFP or (right lanes) HIV Gag(p49)-GFP. The same ratio of exosome lysate:cell lysate was used for both samples. Bar indicate 10 μm.

To identify the budding/exosomal sorting information in HIV Gag, we followed the sorting and secretion of full-length and mutant Gag proteins by Jurkat T cells (human T cells are a primary in vivo host for HIV). HIV Gag-GFP, which buds from cells in a manner similar to that of WT Gag [20], was sorted to ELDs and exosomes (Figure 6B–6I, 6R, 6S, and 6V), as we reported previously [3]. HIV Gag(p49)-GFP lacks the entire p6 domain, including the late domain PTAP motif. HIV Gag(p49)-GFP was also sorted to ELDs (Figure 6J–6Q) and secreted from the cell in exosomes (Figure 6T–6V), and at levels similar to that of full-length HIV Gag-GFP. Thus, neither the HIV late domain nor the entire p6 domain were required for HIV Gag budding/exosomal sorting by Jurkat T cells.

We next tested whether higher-order oligomerization was a primary determinant of HIV Gag budding. HIV Gag contains two major oligomerization domains, one in CA and one in NC, and it is already known that loss of either NC or CA blocks Gag budding [25–30]. HIV Gag(p41)-GFP lacks all NC, p1, and p6 sequences, and thus, lacks the interaction domain of NC but still retains one major oligomerization domain, in the C-terminal half of CA (Figure 7A). HIV Gag(p41)-GFP was not sorted to ELDs (Figure 7B–7E) and was not secreted from the cell in exosomes (Figure 7N, lane 2). However, HIV Gag(p41)-GFP could be redirected to ELDs by addition of a heterologous dimerization domain (a synthetic leucine zipper [LZ]): HIV Gag(p41)-LZ-GFP, was sorted to ELDs and secreted from the cell in exosomes (Figure 7F–7I and 7N–7P) as well as HIV Gag-GFP. Thus, the primary budding/exosomal sorting signal in the NC-p1-p6 region of HIV Gag appears to be the oligomerization domain in NC (also known as the I domain [20]), not the HIV late domain motif, at least in Jurkat T cells. This conclusion is supported by the inability of the p6 domain to rescue the budding/exosomal sorting defects caused by loss of NC-p1-p6 (Figure 7J–7N).

**Figure 7:**
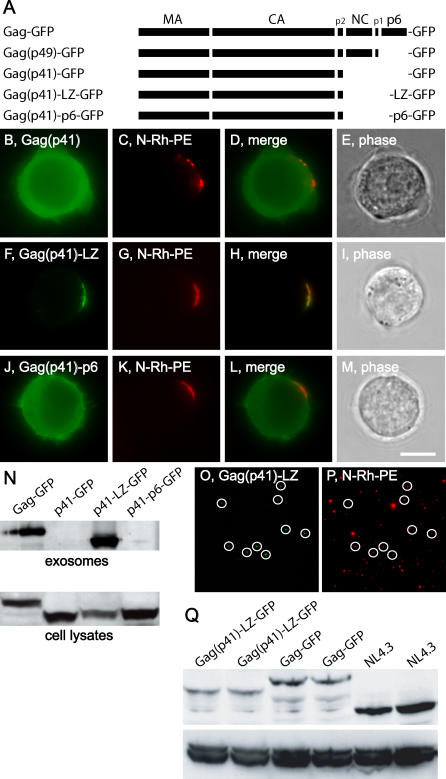
A Synthetic Leucine Zipper Can Suppress the Gag Budding Defect Caused by Loss of NC-p1-p6 (A) Line diagram of full-length HIV Gag and mutant Gag-GFP proteins. (B–M) Fluorescence micrographs of N-Rh-PE–labeled Jurkat T cells expressing (B–E) HIV Gag(p41)-GFP, (F–I) HIV Gag(p41)-LZ-GFP, or (J–M) HIV Gag(p41)-p6-GFP. (N) Anti-Gag immunoblot of exosome and cell lysates of Jurkat T cells expressing (left lanes) full-length HIV Gag-GFP, (center left lanes) HIV Gag(p41)-GFP, (center right lanes) HIV Gag(p41)-LZ-GFP, and (right lanes) HIV Gag(p41)-p6-GFP, all loaded at the same ratio of exosome:cell lysate. (O and P) Fluorescence micrographs of exosomes secreted by N-Rh-PE–labeled Jurkat T cells expressing HIV Gag(p41)-LZ-GFP. White circles mark exosomes that contain both N-Rh-PE and Gag(p41)-LZ-GFP. (Q) Budding of p6-deficient Gag proteins is not an overexpression artifact. Jurkat T cells were transfected twice separately with (left two lanes) pcDNA3/HIVGag(p41)-LZ-GFP, (middle two lanes) pcDNA3/HIVGag-GFP, or (right two lanes) the HIV provirus NL4.3ΔEnv::GFPkdel. Two days later, the cells were lysed, and equal amounts of each lysate were processed for immunoblot using antibodies specific for (upper panel) HIV Gag, and (lower panel) Hsp90 (loading control). Bar indicates 10 μm.

One concern in these experiments is that p6-independent budding of HIV Gag might be an experimental artifact caused by overexpression of HIV Gag. This does not appear to be the case, because control experiments demonstrated that the expression system that we used in our experiments actually drives lower levels of Gag expression in Jurkat T cells than an HIV provirus (Figure 7Q).

We next followed the sorting and secretion of a Gag protein that lacks both of its oligomerization domains. HIV Gag(p39*) is unable to oligomerize or bud from cells, has a point mutation in the CA oligomerization domain (W184A), and lacks all p2, NC, p1, and p6 sequences [26]. As expected, HIV Gag(p39*)-GFP was neither enriched at ELDs nor secreted from the cell in exosomes (Figure 8B–8E and 8V, lane 2). Adding just one oligomerization domain to this protein failed to rescue its exosomal sorting, because neither HIV Gag(p39*)-LZ-GFP nor HIV Gag(p39*)-LZ-DsREDmonomer was sorted to ELDs or exosomes (Figure 8F–8M and 8V, lanes 3 and 4). However, addition of two independent oligomerization domains to HIV Gag(p39*) was sufficient to target HIV Gag(p39*) to ELDs and exosomes. Specifically, we observed that HIV Gag(p39*)-LZ-DsRED, which contains both an oligomeric form of DsRED [38,39] and the dimer-inducing leucine zipper, was sorted to ELDs (Figure 8N–8Q) and secreted from the cell in exosomes (Figure 8V, lane 5, 8W, and 8X) to a similar extent as HIV Gag-DsRED (Figure 8R–8U and 8V). Native gel electrophoresis confirmed that HIV Gag(p39*)-LZ-DsRED exists in a higher oligomeric state than Gag(p39*)-LZ-DsREDm (Figure 8Y).

**Figure 8:**
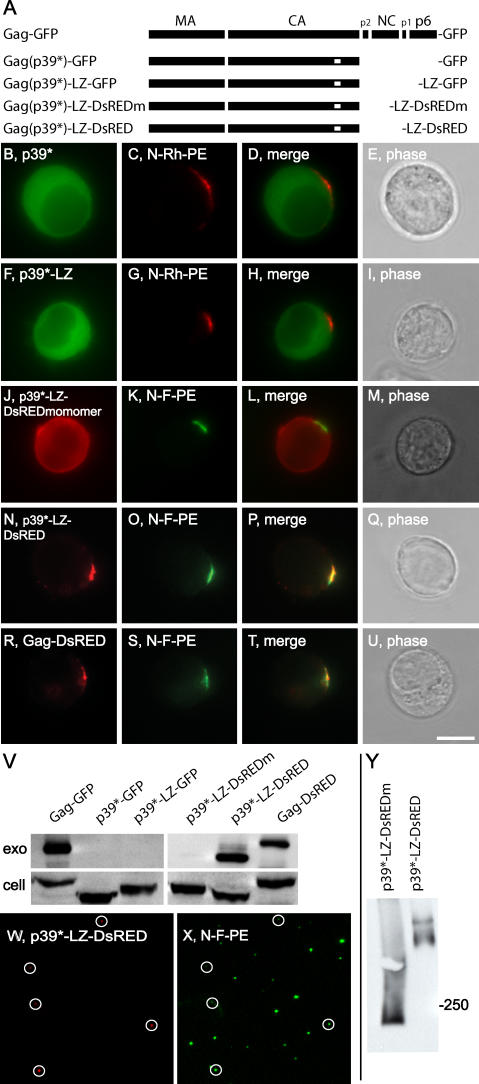
Higher-Order Oligomerization Targets HIV Gag to ELDs and Exosomes (A) Line diagram of full-length HIV Gag and mutant Gag-GFP proteins. (B–I) Fluorescence micrographs of N-Rh-PE–labeled Jurkat T cells expressing (B–E) HIV Gag(p39*)-GFP or (F–I) HIV Gag(p39*)-LZ-GFP. (J–U) Fluorescence micrographs of N-F-PE–labeled Jurkat T cells expressing (J–M) HIV Gag(p39*)-LZ-DsREDmonomer, (N–Q) HIV Gag(p39*)-LZ-DsRED, or (R–U) HIV Gag-DsRED. (V) Anti-Gag immunoblot of exosome (exo) and cell lysates (cell) of Jurkat T cells expressing (lane 1) full-length HIV Gag-GFP, (lane 2) HIV Gag(p39*)-GFP, (lane 3) HIV Gag(p39*)-LZ-GFP, (lane 4) HIV Gag(p39*)-LZ-DsREDmonomer, (lane 5) HIV Gag(p39*)-LZ-DsRED, or (lane 6) HIV Gag-DsRED, all loaded at the same ratio of exosome:cell lysate. (W and X) Fluorescence micrographs of exosomes secreted by N-F-PE–labeled Jurkat T cells expressing HIV Gag(p39*)-LZ-DsRED. White circles mark exosomes that contain both N-F-PE and Gag(p39*)-LZ-DsRED. (Y) Anti-Gag immunoblot of native cell lysates generated from Jurkat T cells expressing (left lane) HIV Gag(p39*)-LZ-DsREDmonomer or (right lane) HIV Gag(p39*)-LZ-DsRED, separated by native gel electrophoresis. Bar indicates 10 μm.

### Creating a Synthetic Exosomal Cargo

The simplest conclusion from these results is that higher-order oligomerization, rather than any specific sequences in HIV Gag, is a primary determinant of HIV Gag budding/exosomal sorting. Such a conclusion, however, fails to account for the fact that other sequences in HIV Gag(p39*) might contribute to Gag budding, such as those that bind AP-3 [40], perhaps phosphatidylinositol-4,5-bisphosphate [41], etc. To address the possibility that these provide a unique and essential foundation for HIV Gag budding/exosomal sorting, we removed all Gag sequences from HIV Gag(p39*)-LZ-DsREDmonomer and HIV Gag(p39*)-LZ-DsRED, and replaced them with a plasma membrane anchor (NH_2_-MGCINSKRKD-COOH [19]). The first of these proteins, Acyl-LZ-DsREDmonomer, showed little or no enrichment at ELDs and was a poor exosomal cargo (Figure 9A–9D, 9I, 9J, and 9M). In contrast, Acyl-LZ-DsRED was efficiently secreted from the cell in exosomes (Figure 9E–9H, 9K, 9L, and 9M). These results demonstrate two important points: first, that higher-order oligomerization and plasma membrane binding are sufficient to target a protein to exosomes, and second, that it is possible to generate a synthetic exosomal cargo based on this principle.

**Figure 9:**
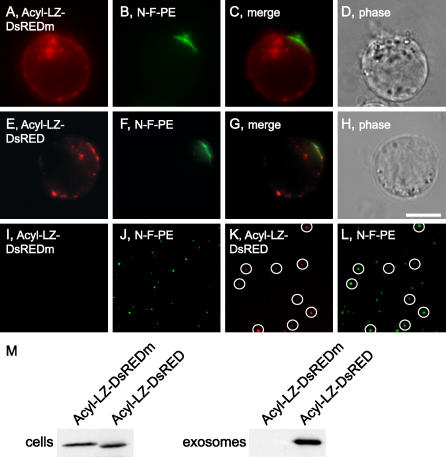
Vesicular Secretion of a Synthetic Exosomal Cargo (A–H) Fluorescence micrographs of N-F-PE–labeled Jurkat T cells expressing (A–D) Acyl-LZ-DsREDmonomer or (E–H) Acyl-LZ-DsRED. (I–L) Fluorescence micrographs of exosomes secreted by N-F-PE–labeled Jurkat T cells expressing (I and J) Acyl-LZ-DsREDmonomer or (K and L) Acyl-LZ-DsRED. White circles mark exosomes that contain both N-F-PE and Acyl-LZ-DsRED. Bar indicates 10 μm. (M) Anti-DsRED immunoblot of exosome and cell lysates of Jurkat T cells expressing (left lanes) Acyl-LZ-DsREDmonomer or (right lanes) Acyl-LZ-DsRED (equal ratios of exosome lysate:cell lysate).

### Relevance to HIV Virus Budding

The p6-independent, oligomerization-induced budding/exosomal secretion of HIV Gag is, from a virological perspective, most important if it also informs our understanding of HIV virus budding. To address this issue, we followed the budding of control and p6-deficient HIV viruses from human cells. It is well established that loss of p6 severely reduces HIV budding from human kidney-derived 293T cells [32,35,36], and we therefore used these cells to assess the role of HIV p6 in HIV virus budding. 293T cells were transfected with a control HIV provirus, NL4.3ΔEnv::GFPkdel (an Env-deleted, but otherwise replication-competent, variant of the HIV provirus NL4.3 [42]), and these cells budded significant levels of virus into the medium (Figure 10A, left lanes). As expected from the literature, 293T cells transfected with a p6-deficient provirus, NL4.3ΔEnv::GFPkdel/p6^L1ter^, budded far less virus (Figure 10A, middle lanes). (The p6^L1ter^ mutant selectively abrogates p6 expression [35].)

**Figure 10:**
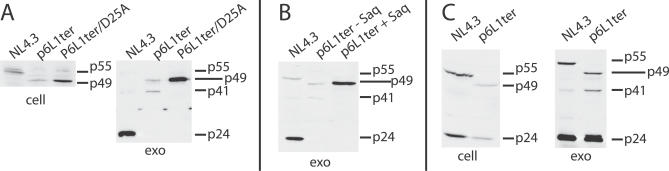
HIV Virus Can Bud from Cells Independently of Its p6 Domain (A) Anti-HIV Gag immunoblots of cell (cell) and exosome (exo) lysates (constant exosome:cell ratio) of 293T cells transfected with equal amounts of (left lanes) pNL4.3ΔEnv::GFPkdel, (middle lanes) pNL4.3ΔEnv::GFPkdel/p6^L1ter^, and (right lanes) pNL4.3ΔEnv::GFPkdel/p6^L1ter^/PR^D25A^. (B) Anti-HIV Gag immunoblots of exosomes secreted by 293T cells transfected with equal amounts of (left lane) pNL4.3ΔEnv::GFPkdel or (middle and right lanes) pNL4.3ΔEnv::GFPkdel/p6^L1ter^ , incubated in the (middle lane) absence or (right lane) presence of 7.5-μm HIV protease inhibitor. (C) Anti-HIV Gag immunoblots of cell and exosome lysates from Jurkat T cells transfected with equal amounts of (left lanes) pNL4.3ΔEnv::GFPkdel and (right lanes) pNL4.3ΔEnv::GFPkdel/p6^L1ter^.

At first glance, these results seem to indicate that the p6-independent budding of HIV Gag might not be very informative for the mechanism of HIV virus budding. However, the HIV provirus expresses several additional proteins, and these have the potential to influence the exosomal sorting of HIV Gag. In particular, HIV protease (PR) is a known inhibitor of HIV budding [43] and a predicted destroyer of exosomal sorting information in HIV Gag (PR cleaves Gag between its matrix [MA], CA, and NC domains [22]). Moreover, Huang et al [44] reported more than a decade ago that loss of the HIV late domain did not impair the budding of a PR-deficient HIV mutant. To test whether the budding defect of HIV late domain mutants is an indirect effect and caused primarily by HIV PR activity, we followed the budding of a p6-deficient, PR-deficient HIV provirus, NL4.3ΔEnv::GFPkdel/p6^L1ter^/PR^D25A^, which has both the p6^L1ter^ mutation and an inactivating mutation [D25A] in PR. This p6-deficient, PR-deficient HIV virus showed no budding defect in 293T cells (Figure 10A, right lanes). Consistent with these results, we observed that the HIV protease inhibitor saquinavir suppressed the budding defect of p6-deficient HIV in 293T cells (Figure 10B). Previous reports have demonstrated that HIV budding is unaffected by PR inhibitors or PR mutations [24,44,45].

These results indicate that the p6 domain, although it plays an important role in HIV virus budding from 293T cells, is unlikely to play a direct, mechanistically essential role in the budding process. This general conclusion is also supported by studies of HIV budding in human T cells. Demirov et al. [35] previously demonstrated that loss of p6, or the HIV late domain alone, “had little or no effect on particle release” from any of several human T cell lines and from primary human leukocytes (peripheral blood mononuclear cells). We repeated these experiments in our own strain of Jurkat T cells and obtained similar results: our control and p6-deficient HIV viruses showed no significant difference in HIV budding from Jurkat T cells (Figure 10C). Taken together, these results indicate that the p6-independent budding of HIV Gag we observed earlier (Figures 6–8) has high relevance for HIV virus budding.

### Class E VPS Function, Exosome Biogenesis, and HIV Budding

The class E VPS proteins are thought to play direct roles in trafficking cargoes to MVBs, the formation of outward budding vesicles, and retrovirus budding [14,31,32]. However, recent studies have demonstrated that inhibition of class E VPS function does not block MVB biogenesis in animal cells [46–48]. To determine whether class E VPS function is required for exosome biogenesis, we took advantage of the fact that expression of an ATPase-defective form of VPS4B impairs class E VPS function [32,37,49]. Specifically, we generated a Jurkat T cell line that expressed DsRED-VPS4B/K180Q from a tetracycline-inducible promoter. The tetracycline-inducible expression of DsRED-VPS4B/K180Q is evident here in the fluorescence micrograph of two cells, one an uninduced cell that had been labeled with the plasma membrane marker PKH-67 (green) and fixed, the other a cell that had been exposed to 10 μg/ml tetracycline overnight, and then fixed (Figure 11A–11C).

**Figure 11:**
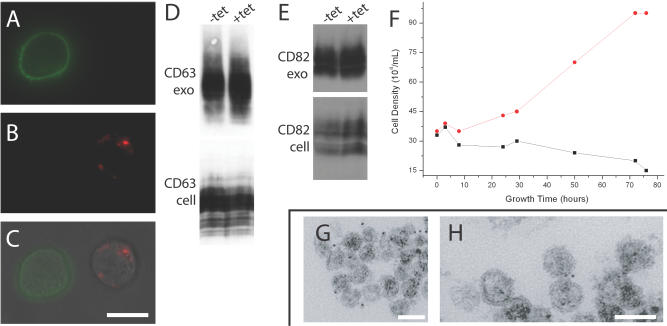
Impairing Class E VPS Function Does Not Block Exosome Budding (A–C) Fluorescence micrographs of T-rex/DsRED-VPS4B/K180Q cells that had been (left cell) grown in the absence of tetracycline, labeled with the green plasma membrane dye PKH-67, and fixed, or (right cell) incubated overnight with tetracycline and fixed. Following fixation, the two cell populations were mixed and images were collected for (A) PKH-67 fluorescence, (B) DsRED-VPS4B/K180Q fluorescence, and (C) phase contrast, which was merged with the two fluorescent images. Bar indicates 10 μm. (D and E) Immunoblots of exosome (exo) and cell lysates generated from T-rex/DsRED-VPS4B/K180Q cells incubated with (+tet) or without (−tet) tetracycline overnight, blotted with antibodies specific for (D) CD63 and (E) CD82. (F) Growth curve (cell density [10^4^ cells/ml] vs. time in hours) of T-rex/DsRED-VPS4B/K180Q cells in the (red circles) absence or (black squares) presence of tetracycline. (G and H) Immunoelectron micrographs of exosomes secreted by K562 cells expressing DsRED-VPS4B/K180Q, labeled with immunogold for the exosomal protein CD63. Bar indicates 100 nm.

Exosomes collected from cells incubated with or without tetracycline had similar levels of the exosomal markers CD63 and CD82 (Figure 11D and 11E), indicating that inhibition of class E VPS function did not block exosome budding or the secretion of these proteins from the cell in exosomes. Class E VPS function is, however, required for cell growth and viability, and addition of tetracycline prevented cell growth (Figure 11F). Exosomes secreted by cells expressing DsRED-VPS4B/K180Q had the size and spheroid morphology expected of exosomes, though they did seem prone to clustering (Figure 11G and 11H).

We next tested whether class E VPS proteins play the direct and essential role in retrovirus budding that is currently favored [14,31]. To explore this issue, we used a system established by Sundquist and colleagues for studying the role of class E VPS function in HIV budding [32,37,49]. This involves co-transfection of human cells with (1) plasmids designed to express WT or ATPase-defective forms of the AAA ATPase VPS4B (DsRED-VPS4B/K180Q and DsRED-VPS4B/E235Q) and (2) HIV proviruses or HIV Gag expression vectors, then measuring the amount of Gag released from the cells in sedimentable particles. Using the same VPS4B-expressing plasmids as Sundquist and colleagues, we too observed that inhibiting class E VPS function impaired the budding of HIV virus, in this case the budding of NL4.3ΔEnv::GFPkdel from Jurkat T cells (Figure 12A). However, inhibiting class E VPS function had no detectable effect on the budding of the p6-deficient HIV virus NL4.3ΔEnv::GFPkdel/p6^L1ter^/PR^D25A^ from Jurkat T cells (Figure 12B).

**Figure 12:**
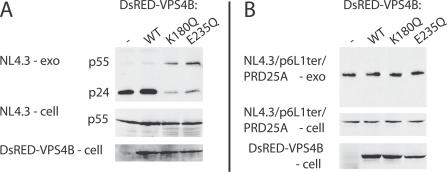
Removal of p6 Allows HIV to Bud Independently of Class E VPS Function Jurkat T cells were co-transfected the HIV proviruses (A) pNL4.3ΔEnv::GFPkdel or (B) pNL4.3ΔEnv::GFPkdel/p6^L1ter^/PR^D25A^, and plasmids designed to express (left lanes) no VPS4B protein (−), (center left lanes) DsRED-VPS4B (WT), (center right lanes) DsRED-VPS4B/K180Q (K180Q), or (right lanes) DsRED-VPS4B/E235Q (E235Q). Two days later, the cells and exosomes were collected, separated by SDS-PAGE (constant ratio of cells:exosomes in all experiments), and processed for immunoblot using antibodies specific for (upper and middle panels) HIV Gag and (lower panel) DsRED.

## Discussion

Targeting signals are the elements in a protein that are sufficient, and not merely necessary, to direct it to its proper location. Here we presented several lines of evidence that higher-order oligomerization and membrane binding are sufficient to target proteins to exosomes. Specifically, we observed that (1) addition of both monoclonal mouse IgG to CD43 and polyclonal anti-mouse IgG antibodies were sufficient to induce the sorting of CD43 to ELDs and exosomes, (2) addition of a plasma membrane anchor was sufficient to target the highly oligomeric cytosolic protein TyA to ELDs and exosomes, (3) a synthetic cargo comprised of a plasma membrane anchor and two heterologous oligomerization domains (Acyl-LZ-DsRED) was sorted to exosomes, (4) highly oligomeric, plasma membrane-associated retroviral Gag proteins (from EIAV, HTLV-1, RSV, MLV, MPMV, and HERV-K) were all sorted to ELDs and exosomes, and (5) a pair of heterologous oligomerization domains was necessary and sufficient to target HIV Gag to ELDs and exosomes.

The finding that higher-order oligomerization is sufficient to target plasma membrane proteins to exosomes is consistent with the known composition of exosomes. Many common exosomal cargoes are known to be components of highly oligomeric, membrane-associated protein complexes, including tetraspanins [50], retroviral Gag proteins [3,12], amyloidogenic proteins [51], and even the class E VPS proteins themselves [52,53]. Our results are also consistent with the results of Vidal et al. [54], who demonstrated that exogenous antibodies enhanced the exosomal sorting of two normally exosomal transmembrane proteins (the transferrin receptor and acetylcholinesterase) from maturing reticulocytes.

The exosomal protein-sorting pathway described here appears to show little or no dependence on the protein's sequence, function, or topology in the plasma membrane. As such, it represents an unusual variation on the general paradigm of protein sorting in which organellar proteins possess relatively short targeting signals [55]. It is unclear how higher-order oligomerization per se could generate a unique peptide-based signal that would be recognized by the presumptive exosomal protein sorting machinery. Therefore, we prefer an indirect hypothesis of cargo selection. For example, higher-order oligomerization of membrane proteins might induce their desolvation, and this might be the cue for cargo selection. An indirect mechanism predicts significant variability in the types of oligomerization domains, membrane anchors, and cargo proteins that are compatible with exosomal cargo selection, as well as significant interplay between protein expression level, inter-subunit affinity, and membrane affinity in determining a protein's exosomal sorting.

As for the mechanism of exosome biogenesis, we expected that class E VPS proteins would play an essential role. This expectation was based on the prevailing hypothesis that class E VPS proteins sort proteins to sites of outward vesicle budding at endosomal membranes, catalyze the biogenesis of outward budding vesicles, and mediate the budding of HIV and other retroviruses [14,31]. However, we found that inhibition of class E VPS function had no demonstrable effect on exosome budding or the vesicular secretion of two known exosomal markers, CD63 and CD82, even while it blocked cell growth. These results indicate that class E VPS function is not a direct, mechanistic, and essential step in exosome biogenesis. These results are consistent with studies of MVB biogenesis in animal cells that have shown that inhibiting class E VPS function does not block MVB biogenesis or the sorting of nonubiquitylated cargoes to MVBs, though it can impair the sorting of some cargoes to MVBs [46–48].

### Implications for Retroviral Biogenesis

Our data indicate that protein sorting to retroviral VLPs and viruses is mediated by the same signals that target proteins to exosomes. Our first evidence for this was the finding that higher-order oligomerization of CD43 is sufficient to induce its trafficking to HIV Gag VLPs. Other parallels between exosomal protein sorting and protein trafficking to VLPs/viruses include the sorting of diverse Gag proteins to ELDs and exosomes, the oligomerization-induced sorting of Gag to ELDs and exosomes, the ability to remove p6 without affecting release of HIV Gag VLPs or release of PR-deficient HIV virus, the fact that adding a plasma membrane anchor was sufficient to induce the budding of TyA (the Gag-like structural protein of a yeast LTR retrotransposon), and the fact that all budding-competent proteins were sorted to ELDs and secreted from the cell in exosomes, whether of viral origin or not. An exosomal origin of HIV is also consistent with many prior observations [11], including the secretion of amyloidogenic, exosomal proteins on retrovirus particles [56] and the interactions between HTLV-1 Gag and the exosomal proteins CD81 and CD82 [57].

The primary significance of these observations is that they reveal retrovirus budding to be a manifestation of a normal, cell-encoded exosome biogenesis pathway. This has important implications for the targeting of viral and nonviral proteins to sites of retrovirus budding and onto retrovirus particles, the identification and characterization of retrovirus budding factors (and exosome biogenesis factors), and potentially for the targeting of antiretroviral agents to sites of budding and onto retroviral particles. An exosomal mechanism of retrovirus budding is also likely relevant to the evolutionary relationships between retroviruses and LTR retrotransposons [11]. For example, the ability to target TyA to ELDs and exosomes merely by adding an acylation tag indicates that acquisition of exosomal sorting information might be a critical step in the evolution of retroviruses from LTR retrotransposons, and that loss of membrane binding might mediate the reverse transition. This notion is consistent with the ability to convert the mouse LTR retrotransposon MusD from an intracellular, noninfectious transposon into a budding, infectious retrovirus merely by appending a retroviral MA domain to the N-terminus of its Gag-like protein [58].

The intracellular trafficking of HIV Gag has been studied closely. Some have concluded that retrovirus assembly initiates at endosomes and then proceeds to completion either there or at the plasma membrane [8,10,59–61], whereas others report that retrovirus assembly initiates at the plasma membrane and proceeds to budding either there or at endosomes [62]. To us, these pathways are all consistent with an exosomal origin for HIV, for they are each compatible with the fact that exosome budding can occur at either ELDs or endosomes. Specifically, it appears that exosomes can bud from either endosomes or from ELDs, and that ELDs might form by lateral sorting in the plasma membrane as well as by endosome–plasma membrane fusion. Our results also have some relevance for exosome morphogenesis. The varied sizes and shapes of AcylTyA-containing exosomes indicate that these characteristics are not under strict mechanistic control. This suggests a model of exosome morphogenesis in which vesicle size and shape are mediated by the cargoes themselves. This would be consistent with the highly uniform morphology of retrovirus particles [13], as well as the pronounced effects that certain Gag and PR mutations can have on virus morphology [24].

An exosomal model of retrovirus budding also offers new insights into why HIV budding is promoted by the HIV late domain. The most parsimonious interpretation of the data is that the late domain promotes virus budding indirectly. As for how, one possibility is that the late domain negatively regulates HIV PR until after budding, thereby ensuring the oligomerization-induced sorting of HIV Gag to exosomes. This hypothesis is supported by additional observations in the literature, such as the inhibitory effect of PR activity on budding [43] and the premature cleavage of the HIV Gag-Pol polyprotein in the context of an HIV late domain mutant [63]. The direct and indirect interactions between the HIV late domain and the class E VPS machinery indicate that they act together. Our data support this notion insofar as they show that class E VPS function appears to promote HIV budding via an indirect mechanism.

It should be noted that our data do not exclude the possibility of more complex retrovirus budding mechanisms. For example, it is formally possible that the p6 domain and class E VPS proteins play direct, mechanistic, and essential roles in delivering HIV Gag to exosomes in some other cell types, or perhaps in a non-exosomal mechanism of budding. However, this last alternative offers no explanation for why HIV budding occurs at the same sites as exosome biogenesis or why HIV particles are enriched in exosomal markers [3,8,9,11,12,56,64–66].

## Materials and Methods

### Reagents and antibodies.

Commercial sources were used for the acquisition of N-Rh-PE, N-F-PE (Avanti Polar Lipids, http://www.avantilipids.com), PKH-67 (Sigma, http://www.sigmaaldrich.com), HIV protease inhibitor saquinavir (Moravek,http://www.moravek.com), and antibodies (Santa Cruz Biotechnology, http://www.scbt.com; Chemicon International, http://www.chemicon.com; Pharmingen, http://www.bdbiosciences.com; Abcam, http://www.abcam.com; and Jackson ImmunoResearch Laboratories, http://www.jacksonimmuno.com). Rabbit anti-HIV Gag p24 antibodies were from James Hildreth (Meharry Medical College, Nashville, Tennessee).

### Plasmids.

HIV Gag-GFP and AIP1/VPS31-DsRED were described previously [3], the WT and mutant VPS4B expression vectors were from Wes Sundquist (University of Utah), pNL4.3ΔEnv::GFPkdel was from Robert Siliciano (Johns Hopkins University), and pDsRed-monomer-N1 was from Clontech (http://www.clontech.com). HIV Gag mutants were amplified using primers designed to append an Asp718 site (GGTACC) immediately upstream of the start codon and a BamHI site (GGATCC) at the 3′ end of the open reading frame (ORF). Following cleavage with Asp718 and BamHI, each PCR product was inserted between the Asp718 and BamHI sites of pcDNA3-GFP, pcDNA3-DsRed, or pcDNA3-DsREDm, in each case generating a continuous ORF encoding an in-frame fusion between the Gag ORF and the fluorescent protein ORF. To generate ORFs encoding LZ fusions, a fragment encoding NH_2_-LQRMKQLEDKVEELLSKNYHLENEVTRLKKLVGE-COOH was inserted between the Gag and fluorescent protein ORFs. Protein sequences for EIAV Gag, HTLV-1 Gag, MLV Gag, RSV Gag, MPMV-Gag, HERV-K Gag, SFV Gag, and TyA were used to design and synthesize human codon-optimized ORFs with Asp718 and BamHI sites flanking the ORF and inserted between the Asp718 and BamHI sites of pcDNA3-GFP. AcylTyA-GFP and Acyl(G2A,C3A)TyA-GFP were generated by amplifying the TyA ORF with primers designed to append codons for NH_2_-MGCINSKRKD-COOH or the G2A,C3A mutant version to the 5′ end of the ORF, which was also cloned upstream of and in frame with the GFP ORF in pcDNA3-GFP. Plasmids pNL4.3ΔEnv::GFPkdel/p6^L1ter^ and pNL4.3ΔEnv::GFPkdel/p6^L1ter^/PR^D25A^ were generated by amplifying the ApaI-SbfI fragment of pNL4.3ΔEnv::GFPkdel with primers designed to introduce the p6^L1ter^ mutation (change of nucleotides 2188–2190 from CTT to TGA) or both the p6^L1ter^ mutation and the D25A mutation of protease (change of nucleotides 2379–2381 from GAT to GCC) and then inserting these fragments into pNL4.3ΔEnv::GFPkdel. All experiments were performed with sequence-confirmed plasmids.

### Cell culture, lipid labeling, transfection, and microscopy.

Jurkat cells and K562 cells (James Hildreth, Meharry Medical College, Nashville, Tennessee) were maintained in serum-free Aim V medium (GIBCO BRL, http://www.invitrogen.com), and our derivative of the Tet-on Jurkat T cell line (Trex; Invitrogen, http://www.invitrogen.com) was maintained in RPMI/10% fetal calf serum, with induction in 1 μg/ml tetracycline. Cells were pulse labeled with N-Rh-PE, N-F-PE, or PKH-67 as described [3]. Surface proteins were oligomerized by incubating cells with mouse monoclonal antibodies (1:100) for 45 min, washing four times with 1× PBS, incubating with secondary antibodies (1:100) for 35 min, washing three more times with 1× PBS, all at 4 °C. Cells were then either fixed or incubated at 37 °C for the time indicated and then fixed. Cells were transfected by mixing 1 × 10^7^ cells with 5–10 μg of plasmid DNA (2:1 ratio for co-transfections) at room temperature for 15 min, followed by electroporation at 300 V, 24 Ω, 800 μF. Images were collected and processed as described [3]. Immunogold surface labeling of cells was performed as described [3,67]. All images showing co-localization of proteins at ELDs were from experiments in which co-localization was detected in the majority of relevant cells, with the sole exception being the experiments with Acyl-LZ-DsRED and RSV Gag-GFP, in which co-localization was detected in approximately 10% of relevant cells.

### Exosome isolation, protease protection, and immunoblots.

Exosomes were collected, analyzed by immunoblot and fluorescence microscopy, and purified by density gradient as described [3]. Protease protection experiments were performed by incubating exosomes with trypsin in the presence or absence of 0.1% Triton X-100. For native gel electrophoresis, cells were lysed in 50 mM Tris HCl (pH 7.5), 150 mM NaCl, 1 mM EDTA, 1% Triton X-100, 1× protease inhibitor cocktail (Boerhinger-Ingelheim, http://www.boehringer-ingelheim.com), rotated at 4 °C for 2 h, clarified by centrifugation at 15,000*×g* for 15 min, separated by electrophoresis through 4%–20% gradient native gels (Invitrogen), and processed for immunoblot.

## Supporting Information

### Accession Numbers

The GenBank (http://www.ncbi.nlm.nih.gov/Genbank) accession numbers for the protein sequences discussed in this paper are as follows: EIAV Gag (M16575), HERV-K Gag (Y17833), HTLV-1 Gag (D13784), MLV Gag (J02255), MPMV-Gag (M12349), RSV Gag (J02342), SFV Gag (U04327), and TyA (M18706). The GenBank accession numbers for the human codon-optimized ORFs are as follows: EIAV Gag (DQ421317), HERV-K Gag (DQ421322), HTLV-1 Gag (DQ421318), MLV Gag (DQ421319), MPMV Gag (DQ421321), RSV Gag (DQ421320), SFV Gag (DQ866825), and TyA (DQ421323).

## Abbreviations

CA: capsid
EIAV: equine infectious anemia virus
ELD: endosome-like domain
FITC: fluorescein isothiocyanate
GFP: green fluorescent protein
HERV-K: human endogenous retrovirus K
HIV: Human immunodeficiency virus
HTLV-1: human T-cell lymphotropic virus
IgG: immunogammaglobulin
LTR: long terminal repeat
LZ: leucine zipper
MLV: murine leukemia virus
MPMV: Mason-Pfizer monkey virus
MVB: multivesicular body
NC: nucleocapsid
ORF: open reading frame
PR: protease
RSV: Rous sarcoma virus
SFV: simian foamy virus
VLP: virus-like particle
VPS: vacuolar protein-sorting
WT: wild-type

## References

[pbio-0050158-b001] Trams EG, Lauter CJ, Salem N, Heine U (1981). Exfoliation of membrane ecto-enzymes in the form of micro-vesicles. Biochim Biophys Acta.

[pbio-0050158-b002] Thery C, Zitvogel L, Amigorena S (2002). Exosomes: Composition, biogenesis and function. Nat Rev Immunol.

[pbio-0050158-b003] Booth AM, Fang Y, Fallon JK, Yang JM, Hildreth JE (2006). Exosomes and HIV Gag bud from endosome-like domains of the T cell plasma membrane. J Cell Biol.

[pbio-0050158-b004] Denzer K, Kleijmeer MJ, Heijnen HF, Stoorvogel W, Geuze HJ (2000). Exosome: From internal vesicle of the multivesicular body to intercellular signaling device. J Cell Sci.

[pbio-0050158-b005] Saez F, Frenette G, Sullivan R (2003). Epididymosomes and prostasomes: Their roles in posttesticular maturation of the sperm cells. J Androl.

[pbio-0050158-b006] Yu X, Harris SL, Levine AJ (2006). The regulation of exosome secretion: A novel function of the p53 protein. Cancer Res.

[pbio-0050158-b007] Gruenberg J, Stenmark H (2004). The biogenesis of multivesicular endosomes. Nat Rev Mol Cell Biol.

[pbio-0050158-b008] Pelchen-Matthews A, Kramer B, Marsh M (2003). Infectious HIV-1 assembles in late endosomes in primary macrophages. J Cell Biol.

[pbio-0050158-b009] Raposo G, Moore M, Innes D, Leijendekker R, Leigh-Brown A (2002). Human macrophages accumulate HIV-1 particles in MHC II compartments. Traffic.

[pbio-0050158-b010] Nydegger S, Foti M, Derdowski A, Spearman P, Thali M (2003). HIV-1 egress is gated through late endosomal membranes. Traffic.

[pbio-0050158-b011] Gould SJ, Booth AM, Hildreth JE (2003). The Trojan exosome hypothesis. Proc Natl Acad Sci U S A.

[pbio-0050158-b012] Nguyen DG, Booth A, Gould SJ, Hildreth JE (2003). Evidence that HIV Budding in primary macrophages occurs through the exosome release pathway. J Biol Chem.

[pbio-0050158-b013] Briggs JA, Johnson MC, Simon MN, Fuller SD, Vogt VM (2006). Cryo-electron microscopy reveals conserved and divergent features of gag packing in immature particles of Rous sarcoma virus and human immunodeficiency virus. J Mol Biol.

[pbio-0050158-b014] Hurley JH, Emr SD (2006). The ESCRT complexes: Structure and mechanism of a membrane-trafficking network. Annu Rev Biophys Biomol Struct.

[pbio-0050158-b015] Raff MC, De Petris S (1973). Movement of lymphocyte surface antigens and receptors: The fluid nature of the lymphocyte plasma membrane and its immunological significance. Fed Proc.

[pbio-0050158-b016] Schreiner GF, Unanue ER (1976). Membrane and cytoplasmic changes in B lymphocytes induced by ligand-surface immunoglobulin interaction. Adv Immunol.

[pbio-0050158-b017] Roth JF (2000). The yeast Ty virus-like particles. Yeast.

[pbio-0050158-b018] Boeke JD, Eichinger D, Castrillon D, Fink GR (1988). The Saccharomyces cerevisiae genome contains functional and nonfunctional copies of transposon Ty1. Mol Cell Biol.

[pbio-0050158-b019] Zacharias DA, Violin JD, Newton AC, Tsien RY (2002). Partitioning of lipid-modified monomeric GFPs into membrane microdomains of live cells. Science.

[pbio-0050158-b020] Sandefur S, Varthakavi V, Spearman P (1998). The I domain is required for efficient plasma membrane binding of human immunodeficiency virus type 1 Pr55Gag. J Virol.

[pbio-0050158-b021] Coffin JM, Hughes SH, Varmus HE (1997). Retroviruses.

[pbio-0050158-b022] Freed EO, Martin MA, Knipe DM, Howley PM (2001). HIVs and their replication. Fields virology.

[pbio-0050158-b023] Briggs JA, Simon MN, Gross I, Krausslich HG, Fuller SD (2004). The stoichiometry of Gag protein in HIV-1. Nat Struct Mol Biol.

[pbio-0050158-b024] Gottlinger HG, Sodroski JG, Haseltine WA (1989). Role of capsid precursor processing and myristoylation in morphogenesis and infectivity of human immunodeficiency virus type 1. Proc Natl Acad Sci U S A.

[pbio-0050158-b025] Accola MA, Strack B, Gottlinger HG (2000). Efficient particle production by minimal Gag constructs which retain the carboxy-terminal domain of human immunodeficiency virus type 1 capsid-p2 and a late assembly domain. J Virol.

[pbio-0050158-b026] Burniston MT, Cimarelli A, Colgan J, Curtis SP, Luban J (1999). Human immunodeficiency virus type 1 Gag polyprotein multimerization requires the nucleocapsid domain and RNA and is promoted by the capsid-dimer interface and the basic region of matrix protein. J Virol.

[pbio-0050158-b027] Ganser BK, Li S, Klishko VY, Finch JT, Sundquist WI (1999). Assembly and analysis of conical models for the HIV-1 core. Science.

[pbio-0050158-b028] Ganser-Pornillos BK, von Schwedler UK, Stray KM, Aiken C, Sundquist WI (2004). Assembly properties of the human immunodeficiency virus type 1 CA protein. J Virol.

[pbio-0050158-b029] Spearman P, Wang JJ, Vander Heyden N, Ratner L (1994). Identification of human immunodeficiency virus type 1 Gag protein domains essential to membrane binding and particle assembly. J Virol.

[pbio-0050158-b030] Sandefur S, Smith RM, Varthakavi V, Spearman P (2000). Mapping and characterization of the N-terminal I domain of human immunodeficiency virus type 1 Pr55(Gag). J Virol.

[pbio-0050158-b031] Morita E, Sundquist WI (2004). Retrovirus budding. Annu Rev Cell Dev Biol.

[pbio-0050158-b032] Garrus JE, von Schwedler UK, Pornillos OW, Morham SG, Zavitz KH (2001). Tsg101 and the vacuolar protein sorting pathway are essential for HIV-1 budding. Cell.

[pbio-0050158-b033] Strack B, Calistri A, Craig S, Popova E, Gottlinger HG (2003). AIP1/ALIX is a binding partner for HIV-1 p6 and EIAV p9 functioning in virus budding. Cell.

[pbio-0050158-b034] Huang CH, Reid ME, Xie SS, Blumenfeld OO (1996). Human red blood cell Wright antigens: A genetic and evolutionary perspective on glycophorin A-band 3 interaction. Blood.

[pbio-0050158-b035] Demirov DG, Orenstein JM, Freed EO (2002). The late domain of human immunodeficiency virus type 1 p6 promotes virus release in a cell type-dependent manner. J Virol.

[pbio-0050158-b036] Gottlinger HG, Dorfman T, Sodroski JG, Haseltine WA (1991). Effect of mutations affecting the p6 gag protein on human immunodeficiency virus particle release. Proc Natl Acad Sci U S A.

[pbio-0050158-b037] von Schwedler UK, Stuchell M, Muller B, Ward DM, Chung HY (2003). The protein network of HIV budding. Cell.

[pbio-0050158-b038] Sacchetti A, Subramaniam V, Jovin TM, Alberti S (2002). Oligomerization of DsRed is required for the generation of a functional red fluorescent chromophore. FEBS Lett.

[pbio-0050158-b039] Wall MA, Socolich M, Ranganathan R (2000). The structural basis for red fluorescence in the tetrameric GFP homolog DsRed. Nat Struct Biol.

[pbio-0050158-b040] Dong X, Li H, Derdowski A, Ding L, Burnett A (2005). AP-3 directs the intracellular trafficking of HIV-1 Gag and plays a key role in particle assembly. Cell.

[pbio-0050158-b041] Ono A, Ablan SD, Lockett SJ, Nagashima K, Freed EO (2004). Phosphatidylinositol (4,5) bisphosphate regulates HIV-1 Gag targeting to the plasma membrane. Proc Natl Acad Sci U S A.

[pbio-0050158-b042] Zhang H, Zhou Y, Alcock C, Kiefer T, Monie D (2004). Novel single-cell-level phenotypic assay for residual drug susceptibility and reduced replication capacity of drug-resistant human immunodeficiency virus type 1. J Virol.

[pbio-0050158-b043] Karacostas V, Wolffe EJ, Nagashima K, Gonda MA, Moss B (1993). Overexpression of the HIV-1 gag-pol polyprotein results in intracellular activation of HIV-1 protease and inhibition of assembly and budding of virus-like particles. Virology.

[pbio-0050158-b044] Huang M, Orenstein JM, Martin MA, Freed EO (1995). p6Gag is required for particle production from full-length human immunodeficiency virus type 1 molecular clones expressing protease. J Virol.

[pbio-0050158-b045] Schatzl H, Gelderblom HR, Nitschko H, von der Helm K (1991). Analysis of non-infectious HIV particles produced in presence of HIV proteinase inhibitor. Arch Virol.

[pbio-0050158-b046] Sevrioukov EA, Moghrabi N, Kuhn M, Kramer H (2005). A mutation in dVps28 reveals a link between a subunit of the endosomal sorting complex required for transport-I complex and the actin cytoskeleton in *Drosophila*. Mol Biol Cell.

[pbio-0050158-b047] Theos AC, Truschel ST, Tenza D, Hurbain I, Harper DC (2006). A lumenal domain-dependent pathway for sorting to intralumenal vesicles of multivesicular endosomes involved in organelle morphogenesis. Dev Cell.

[pbio-0050158-b048] Shim JH, Xiao C, Hayden MS, Lee KY, Trombetta ES (2006). CHMP5 is essential for late endosome function and down-regulation of receptor signaling during mouse embryogenesis. J Cell Biol.

[pbio-0050158-b049] Scott A, Chung HY, Gonciarz-Swiatek M, Hill GC, Whitby FG (2005). Structural and mechanistic studies of VPS4 proteins. EMBO J.

[pbio-0050158-b050] Min G, Wang H, Sun TT, Kong XP (2006). Structural basis for tetraspanin functions as revealed by the cryo-EM structure of uroplakin complexes at 6-A resolution. J Cell Biol.

[pbio-0050158-b051] Fevrier B, Vilette D, Archer F, Loew D, Faigle W (2004). Cells release prions in association with exosomes. Proc Natl Acad Sci U S A.

[pbio-0050158-b052] Thery C, Boussac M, Veron P, Ricciardi-Castagnoli P, Raposo G (2001). Proteomic analysis of dendritic cell-derived exosomes: a secreted subcellular compartment distinct from apoptotic vesicles. J Immunol.

[pbio-0050158-b053] Pisitkun T, Shen RF, Knepper MA (2004). Identification and proteomic profiling of exosomes in human urine. Proc Natl Acad Sci U S A.

[pbio-0050158-b054] Vidal M, Mangeat P, Hoekstra D (1997). Aggregation reroutes molecules from a recycling to a vesicle-mediated secretion pathway during reticulocyte maturation. J Cell Sci.

[pbio-0050158-b055] Blobel G (2000). Protein targeting (Nobel lecture). Chembiochem.

[pbio-0050158-b056] Leblanc P, Alais S, Porto-Carreiro I, Lehmann S, Grassi J (2006). Retrovirus infection strongly enhances scrapie infectivity release in cell culture. EMBO J.

[pbio-0050158-b057] Mazurov D, Heidecker G, Derse D (2007). The inner loop of tetraspanins CD82 and CD81 mediates interactions with HTLV-1 gag protein. J Biol Chem.

[pbio-0050158-b058] Ribet D, Harper F, Dewannieux M, Pierron G, Heidmann T (2007). The murine MusD retrotransposon: Structure and molecular evolution of an “intracellularized” retrovirus. J Virol.

[pbio-0050158-b059] Basyuk E, Galli T, Mougel M, Blanchard JM, Sitbon M (2003). Retroviral genomic RNAs are transported to the plasma membrane by endosomal vesicles. Dev Cell.

[pbio-0050158-b060] Perlman M, Resh MD (2006). Identification of an intracellular trafficking and assembly pathway for HIV-1 gag. Traffic.

[pbio-0050158-b061] Sherer NM, Lehmann MJ, Jimenez-Soto LF, Ingmundson A, Horner SM (2003). Visualization of retroviral replication in living cells reveals budding into multivesicular bodies. Traffic.

[pbio-0050158-b062] Jouvenet N, Neil SJ, Bess C, Johnson MC, Virgen CA (2006). Plasma membrane is the site of productive HIV-1 particle assembly. PLoS Biol.

[pbio-0050158-b063] Yu XF, Dawson L, Tian CJ, Flexner C, Dettenhofer M (1998). Mutations of the human immunodeficiency virus type 1 p6Gag domain result in reduced retention of Pol proteins during virus assembly. J Virol.

[pbio-0050158-b064] Nydegger S, Khurana S, Krementsov DN, Foti M, Thali M (2006). Mapping of tetraspanin-enriched microdomains that can function as gateways for HIV-1. J Cell Biol.

[pbio-0050158-b065] Chertova E, Chertov O, Coren LV, Roser JD, Trubey CM (2006). Proteomic and biochemical analysis of purified human immunodeficiency virus type 1 produced from infected monocyte-derived macrophages. J Virol.

[pbio-0050158-b066] Trubey CM, Chertova E, Coren LV, Hilburn JM, Hixson CV (2003). Quantitation of HLA class II protein incorporated into human immunodeficiency type 1 virions purified by anti-CD45 immunoaffinity depletion of microvesicles. J Virol.

[pbio-0050158-b067] McCaffery JM, Farquhar MG (1995). Localization of GTPases by indirect immunofluorescence and immunoelectron microscopy. Methods Enzymol.

